# Identification of a novel microdeletion at 9q21.13 in a family with epilepsy, intellectual disability, and speech disorders and literature review

**DOI:** 10.3389/fgene.2025.1616005

**Published:** 2025-07-07

**Authors:** Liqing Jiang, Jiaqi Li, Aizhong Han, Fei Hou, Xiaotong Wei, Yanjun Tian

**Affiliations:** ^1^ Affiliated Hospital of Jining Medical University (School of Clinical Medicine), Jining Medical University, Jining, Shandong, China; ^2^ Medical Laboratory of Jining Medical University, Jining Medical University, Jining, Shandong, China; ^3^ The Key Laboratory of Multidisciplinary Molecular Diagnosis and Precision Medicine in Medicine and Health of Shandong Province, Jining, Shandong, China

**Keywords:** copy number variations, 9q21.13 microdeletion, epilepsy, intellectual disability, whole exome sequencing, chromosome microarray analysis

## Abstract

**Background:**

At present, there are few reports on 9q21.13 microdeletion syndrome, which is characterized by intellectual disability, epilepsy, autistic behaviour, and recognizable facial features, etc. The aim of this study is to enrich the phenotypic features of 9q21.13 microdeletion syndrome and expand the possible segments of 9q21.13 microdeletion syndrome.

**Methods:**

Four individuals from a 3-generation Chinese family with epilepsy, intellectual disability, and speech disorders were recruited in this study. Whole exome sequencing (WES) and chromosome microarray analysis (CMA) techniques were used for genetic testing. The pathogenicity of CNVs was interpreted following the American College of Medical Genetics (ACMG) standards and guidelines.

**Results:**

A 9q21.13 microdeletion with a fragment size of approximately 2.35 Mb was identified in the proband, the proband’s mother and grandmother and even the fetus. And this region encompasses 6 protein coding genes, namely, *ALDH1A1*, *ANXA1*, *GDA*, *RORB*, *TMC1*, and *ZFAND5*.

**Conclusion:**

In this article, we report a girl with epilepsy, intellectual disability, speech disorders, delayed motor development, and autism. We identified a novel 9q21.13 microdeletion with a fragment size of approximately 2.35 Mb in 4 individuals from a 3-generation Chinese family by WES and CMA techniques. Within the region, the *RORB* gene is a strong candidate gene for complex neurodevelopmental disorders. Herein, we speculate that *RORB* makes a significant contribution to the clinical phenotypes caused by 9q21.13 microdeletion.

## 1 Introduction

Copy number variations (CNVs) are often a significant cause of neurodevelopmental disorders, including autism spectrum disorders, schizophrenia, intellectual disability, developmental delay, epilepsy, etc. Epilepsy is one of the most common neurological disorders characterized by recurrent seizures due to excessive excitation of neurons, with approximately 70% of cases determined by genetic factors (Thomas and Berkovic, 2014). According to reports, 5%–12.7% of epilepsy patients have CNVs (Coppola et al., 2019; Orsini et al., 2018). At present, there are few reports on 9q21.13 microdeletion syndrome, which is characterized by severe developmental delay, epilepsy, neurobehavioral disorders, and recognizable facial features such as hypertelorism, long philtrum, and thin upper lip. There is no unified standard for fragment size in existing reports. Moreover, there is no clear syndrome record in the CNV Syndromes and GeneReviews of DECIPHER (Database of Chromosomal Imbalance and Phenotype in Humans Using Ensembl Resources) database. Boudry-Labis et al. (Boudry-Labis et al., 2013) described the 750 Kb minimal overlapping deletion region in the 9q21.13 locus, which contains 4 genes (*RORB*, *TRPM6*, *NMRK1*, *OSTF1*) and 2 open reading frames (*C9orf40*, *C9orf41*). Among these genes, *RORB* is a strong candidate gene for neurological phenotypes.

The *RORB* (RAR-related orphan receptor B) (OMIM *601972) gene which encodes RORβ, having 2 differentially expressed subtypes, namely, RORβ1 and RORβ2 (André et al., 1998). In humans, RORβ1 is mainly expressed in the cortex, spinal cord, and pituitary gland (Baglietto et al., 2014), while RORβ2 is mainly expressed in the retina and pineal gland (Lal et al., 2015). Jabaudon et al. (Jabaudon et al., 2012) demonstrated that the expression level of RORβ controls the cellular structural pattern of neocortex neurons during development. In addition, it has been reported that RORβ is specifically expressed in cortical samples from patients with temporal lobe epilepsy (Rossini et al., 2011). Rudolf et al. observed that nonsense and missense variants, as well as CNVs of various sizes in the *RORB* gene, may lead to *RORB* haploinsufficiency (HI), resulting in common phenotypic features such as intellectual disability and systemic epilepsy (Rudolf et al., 2016).

In this article, we identified a novel 9q21.13 microdeletion containing the *RORB* gene with a fragment size of approximately 2.35 Mb. The microdeletion is stably inherited in 4 individuals from a 3-generation Chinese family and shares the similar clinical phenotypes as the reported 9q21.13 microdeletion syndrome. The aim of this study is to enrich the phenotypic features of 9q21.13 microdeletion syndrome and expand the possible segments of 9q21.13 microdeletion syndrome.

## 2 Materials and methods

### 2.1 Participants

Four individuals from a 3-generation Chinese family with epilepsy, intellectual disability, and speech disorders were recruited in this study. The clinical phenotypes, comprehensive medical history, and relevant examination results of all affected individuals were documented in detail. The study was reviewed and approved by the Ethics Committee of Affiliated Hospital of Jining Medical University (Ethics Number: 2025-01-C026). Written informed consent was signed by the proband’s parents.

### 2.2 Specimen collection and genomic DNA extraction

5 mL EDTA-anticoagulated peripheral blood was collected from the proband, the parents and the grandmother who visited the Genetic Counseling Clinic of Affiliated Hospital of Jining Medical University on 25 November 2024. Then genomic DNA was obtained from 200 μL of blood via magnetic bead-based extraction method through a Nucleic Acid Extraction Kit (BGI, China) following the manufacturer’s instructions.

The mother underwent amniocentesis at 26 weeks of gestation. The DNA of amniocytes was extracted from 10 mL amniotic fluid using Genomic DNA Extraction Kit (Centrifugal Column Type, TIANGEN, China) in accordance with the operating instructions.

The concentration of genomic DNA was measured by Qubit 4 fluorometer (Thermo Fisher Scientific, United States) according to the manufacturer’s operation manual.

### 2.3 Whole exome sequencing and data analysis

DNA libraries were prepared using a SY-Exome Library Construction Kit (BGI, China). The quality-qualified libraries were pooled in some 0.2 mL PCR tubes. Then exome capture was performed with a SY-Exome Hybridization Capture Kit (BGI, China) according to the manufacturer’s protocols. After making DNA NanoBalls (DNBs), the DNBs were loaded into a sequencing chip for pair-end (100 bp) sequencing using a Universal Reaction Kit for Sequencing (Combinatorial Probe-Anchor Synthesis Sequencing Method) on MGISEQ-2000 platform (BGI, China). Fragment sizes of DNA libraries were detected by bio-fragment analyzer Qsep1 (Bioptic, China). The concentrations of dsDNA (double-stranded DNA) and ssDNA (single-stranded DNA) were measured by Qubit 4 fluorometer (Thermo Fisher Scientific, United States).

The sequencing fragments were aligned to the human reference genome (GRCh37/hg19) by using the Burrows-Wheeler Aligner (BWA). Base quality values of single nucleotide variations (SNVs), insertions/deletions (INDELs), and genotypes were corrected via Genome Analysis Tool Kit (GATK). CNVs at the exon level were detected by ExomeDepth. The use of evidence items and pathogenicity calculation method refered to American College of Medical Genetics (ACMG) standards and guidelines (Riggs et al., 2020). Many databases and prediction softwares were applied to the analysis process, such as 1,000 Genomes, Genome Aggregation Database (gnomAD, https://gnomad.broadinstitute.org/), Exome Aggregation Consortium (ExAC, http://exac.broadinstitute.org/), BGI-Phoenix genetic database (BPGD), SpliceAI and SIFT, etc.

### 2.4 Chromosome microarray analysis and data analysis

Chromosome microarray analysis (CMA) was performed to validate samples with CNVs by whole exome sequencing (WES). Genomic DNA underwent a series of reactions including enzymatic digestion, ligation, polymerase chain reaction (PCR), and fragmentation, resulting in fragments of approximately 25-125 bp. Then, the fragments were labeled with biotin and hybridized with probes on the Affymetrix Cytoscan 750 K chip (Life Technologies, United States). Subsequently, the hybrid chips were cleaned, stained, and analyzed after scanning the fluorescence signals.

The Chromosome Analysis Suite (ChAS) software was utilized to analyze the fluorescence signals in order to identify the samples’ variations in the whole genome range. The pathogenicity of CNVs was interpreted following the ACMG standards and guidelines (Riggs et al., 2020), with reference to databases including but not limited to Database of Genomics Variations (DGV, http://dgv.tcag.ca/dgv), Online Mendelian Inheritance in Man (OMIM, https://omim.org/), DECIPHER (https://decipher.sanger.ac.uk/), ClinGen (https://www.ncbi.nlm.nih.gov/projects/dbvar/clingen/) and PubMed (https://www.ncbi.nlm.nih.gov/pubmed/).

## 3 Results

### 3.1 Clinical phenotypes and family history

The proband was a 7-year-old girl with intellectual disability, epilepsy and autism. The proband developed high muscle tone 2 months after birth and was diagnosed with epilepsy at the age of 2. She was treated with sodium valproate solution for over 1 year, during which she had one seizure. Later, she received antiepileptic treatment with topiramate tablets. At the age of over 2, she could call out “dad” and “mom”, but later she couldn't speak and take care of herself in defecating and urinating. She had delayed motor development, could only walk with a swaying gait, couldn't jump and run fast. She was diagnosed with autism at the age of over 2.

The proband’s mother was 34 years old with slightly lower intelligence. In July 2024, abnormal electromagnetic waves were detected via electroencephalogram, without epileptic seizures and medication treatment. In 2015, she gave birth naturally to a healthy daughter who lives with her ex-husband. The proband was born to her and her current husband in 2017. Her mother (the proband’s grandmother) had intellectual disability, and her father (the proband’s grandfather) had physical disabilities with unknown specific reasons. At 26 weeks of gestation, she underwent amniocentesis. Ultrasound displayed normal fetal development.

Besides, the proband’s father was 37 years old and had a normal phenotype.

### 3.2 Findings of WES

The sequencing depth not less than 200X and whole exome region coverage greater than or equal to 99.5% are quality control metrics in WES. And in this case, the specific sequencing depth and coverage of the proband, the mother and the father were (300.65X, 99.99%), (341.12X, 99.99%) and (293.37X, 99.99%), respectively. The WES showed that the proband and her mother were detected with similar LargeCNV, which were 46,XX,del(9q21.13).seq[GRCh37/hg19](74970868–77230015)*1 and 46,XX,del(9q21.13).seq[GRCh37/hg19](74866254–77112899)*1, respectively. The number of LargeCNV calls of the proband made by ExomeDepth is 29, of which, 9 containing the OMIM genes. And the number of LargeCNV calls of the mother is 31, with 13 containing the OMIM genes. By combining the patients’ clinical phenotypes and CNV pathogenicity, we identified the 9q21.13 deletion. The proband was found to have a deletion of approximately 2.26 Mb in the 9q21.13 region, which contains 5 protein coding genes, namely, *ALDH1A1* (aldehyde dehydrogenase 1 family, member A1, OMIM *100640), *ANXA1* (annexin A1, OMIM *151690), *RORB*, *TMC1* (transmembrane channel-like 1, OMIM *606706), *ZFAND5* (zinc finger, AN1-type domain 5, OMIM *604761). The proband’s mother was found to have a deletion of approximately 2.25 Mb in the 9q21.13 region, which contains 6 protein coding genes, namely, *ALDH1A1*, *ANXA1*, *GDA* (guanine deaminase, OMIM *139260), *RORB*, *TMC1* and *ZFAND5*.

### 3.3 Discoveries of CMA and CNV classification

**FIGURE 1:**
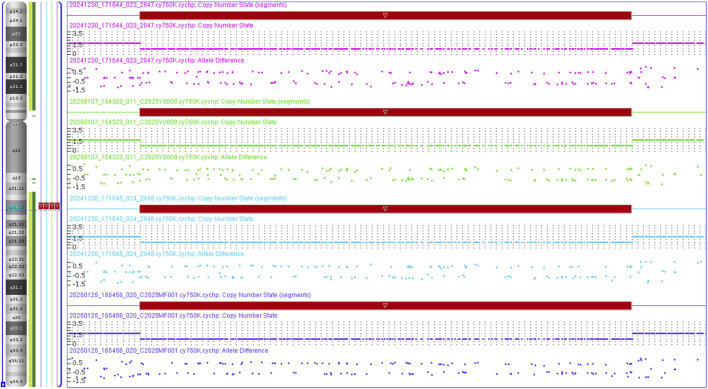
CNV results of the affected family in 9q21.13. The proband, the fetus, the mother and the grandmother were all single copy in 9q21.13. The pink, green, blue, purple colors represent the proband, the fetus, the mother and the grandmother, respectively.

CMA revealed a microdeletion of approximately 2.35 Mb in the 9q21.13 region (arr[GRCh37] 9q21.13(74870591–77222652)x1) in both of the proband, her mother and grandmother, as well as the fetus (Figure 1). It should be noted that the chromosome coordinate has been lifted over in GRCh37 from GRCh38 (arr[GRCh38] 9q21.13(72255,675-74607736)x1) (LiftOver from USCS website, https://genome.ucsc.edu/cgi-bin/hgLiftOver accessed on 21 January 2025). And this microdeletion contains 6 protein coding genes, namely, *ALDH1A1*, *ANXA1*, *GDA*, *RORB*, *TMC1* and *ZFAND5*. Of which, *RORB* and *TMC1* are OMIM Morbid genes, and there are sufficient evidence for HI of *RORB* with a HI score of 3, which is evaluated by ClinGen database on 14 January 2025 (https://search.clinicalgenome.org/kb/gene-dosage/HGNC:10259).

There were no CNVs that fully covered this area in the DGV database, and this CNV has never been reported in the ISCA (International Standards for Cytogenomic Arrays), DECIPHER, and ClinVar databases. A related report on the 9q21.13 microdeletion is retrieved from the PubMed database, which is wholly included in the fragment we detected, with a size of 582 Kb encompassing exon 1 of *RORB* (9q21.13, chr9:76601085-77182821, NM_006914, hg19) in a male patient with childhood absence epilepsy and intellectual disability, while the inheritance of the variant is unknown (Lal et al., 2015). This microdeletion we detected was assessed as a pathogenic CNV (score 1) according to the ACMG guidelines (Riggs et al., 2020), since this CNV contains protein-coding or other known functionally important elements (1A criteria) and partialy overlaps with the 5′ end of an established HI gene and coding sequence is involved (2C-1 criteria), and the number of protein-coding RefSeq genes wholly or partially included in the CNV region is between 0 and 24 (3A criteria), and reported proband has a highly specific phenotype consistent with the gene/genomic region, but the inheritance of the variant is unknown (4E criteria).

## 4 Discussion

**FIGURE 2:**
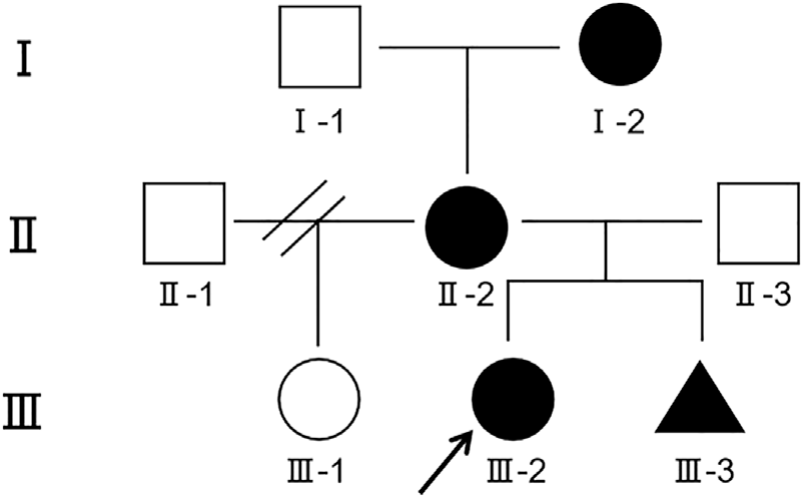
Segregation of 9q21.13 microdeletion in the affected family. The grandmother (Ⅰ-2), the mother (Ⅱ-2), the proband (Ⅲ-2) and even the fetus (Ⅲ-3) were detected with 9q21.13 microdeletion. Circle represents female, square represents male, and the triangle represents gender unknown. Symbols in black indicate the presence of 9q21.13 microdeletion; empty symbols represent unaffected individuals; the arrow represents the proband.

Here we report on a girl with a 2.35 Mb microdeletion at 9q21.13 inherited from her mother and grandmother, with clinical phenotypes of epilepsy, intellectual disability, speech impairment, delayed motor development, and autism. Unfortunately, the fetus in the mother’s womb also had microdeletion in the same region. Intuitive family history is presented in the pedigree chart (Figure 2).

Although the deletion loci are consistent, the clinical phenotypes of family members are not completely the same. The grandmother has the lightest symptoms, only showing intellectual disability. The mother has slightly lower intelligence, and the electroencephalogram shows epileptic waves, but there are no epileptic seizures. While the proband has the most severe symptoms and an early onset age. Different individuals exhibit significant differences in the same variation. Not only in this study, but other studies have also shown significant differences in clinical phenotype and age of onset among individuals with the same variation.

In 2012, the first case with 9q21.13 microdeletion syndrome was identified by Bartnik et al., which is characterized by epilepsy with eyelid myoclonia, generalised tonic-clonic seizures and autism (Bartnik et al., 2012). By comparing the genotypes and phenotypes of 13 patients, Boudry Labis found that these patients share common major clinical features, including mental retardation, developmental delay, epilepsy, neurobehavioral disorders, and characteristic facial features (Boudry-Labis et al., 2013). 13 cases have a minimum overlapping deletion region of 750 Kb, including 4 genes (*RORB*, *TRPM6*, *NMRK1*, *OSTF1*) and 2 open reading frames (*C9orf40*, *C9orf41*). Notably, *RORB* is lost in all 13 cases, which further confirms that *RORB* is a strong candidate for neural phenotypes (Boudry-Labis et al., 2013). From PubMed database, only one case with a microdeletion fully included in our patients’ deletion was selected. The patient has a deletion of 582 Kb on 9q21.13 only encompassing exon 1 of *RORB*, with symptoms of childhood absence epilepsy, intellectual disability, and behavioral problems with aggressive features (Lal et al., 2015; Rudolf et al., 2016). In 2015, Genesio et al. reported a case of 9q21.13 microdeletion syndrome caused by chromothripsis characterized by severe intellectual disability, epilepsy, global developmental delay, dysregulation of platelet aggregation, dysmorphisms, genitalia malformations and hypothyroidism (Genesio et al., 2015). In another case, Tuğ et al. identified a new interstitial deletion within 9q21.11q21.32 confirmed by array comparative genomic hybridization (aCGH) (Tuğ et al., 2018). The patient has many typical features, including but not limited to intellectual disability, development delay, speech disorder, epilepsy, autistic behaviour/behavioral problems as well as craniofacial dysmorphic features (Tuğ et al., 2018). In 2023, a 7-year-old boy with a *de novo* deletion on 9q21.13 was described, presenting global developmental delay, intellectual disability, autistic behaviour, seizures and facial dysmorphism (De et al., 2023).

Apart from the PubMed database, we also searched 31 patients with the deletion in 9q21.13 locus overlapping with our patients’ microdeletion from DECIPHER database. Among them, Boudry-Labis’ 13 patients are included. Meanwhile, we excluded 4 cases without clinical information (291952, 374605, 385645, 501796). The detailed information of the remaining 14 cases is shown in Table 1.

**TABLE 1:** Cases affected by 9q21.13 microdeletion from literatures and DECIPHER.

Source	Patients (35)	Genomic position of deletion on chromosome 9 (GRCh37/hg19)	Deletion size (Mb)	Decipher patient	Age at diagnosis	Sex	Clinical phenotypes
Bartnik et al. (2012)	1	74741400–77306932*	2.57	-	2-year-old	M	Epilepsy with eyelid myoclonia, generalised tonic-clonic seizures and autism
Boudry-Labis et al. (2013)	13	73920074–79528971 (#1)	5.61	250158	8-year-old	M	Mental retardation, speech delay, developmental retardation, epilepsy, autistic behaviour, abnormal brain MRI, facial dysmorphy, hypertrichosis
		73588788–80076668 (#2)	6.49	250165	8-year-old	M	Mental retardation, speech delay, developmental retardation, autistic behaviour, height-weight delay, facial dysmorphy, ocular abnormalities, hypertrichosis
		72182955–79312306 (#3)	7.13	254973	15-year-old	M	Mental retardation, speech delay, developmental retardation, epilepsy, autistic behaviour, hyperactivity, height-weight delay, facial dysmorphy
		73661807–83532389 (#4)	9.87	253847	2-year-old	M	Mental retardation, speech delay, developmental retardation, autistic behaviour, hyperactivity, height-weight delay, facial dysmorphy, prenatal abnormalities
		77047469–79291332 (#5)	2.24	250142	12-year-old	M	Mental retardation, developmental retardation, epilepsy, autistic behaviour, hyperactivity, slight hippocampal asymmetry, facial dysmorphy
		74391472–85348840 (#6)	10.96	250392	6-year-old	F	Mental retardation, speech delay, developmental retardation, epilepsy, autistic behaviour, hyperactivity, abnormal brain MRI, facial dysmorphy, ocular abnormalities, hypertrichosis
		77058421–79277191 (#7)	2.29	261683	16-year-old	M	Mental retardation, speech delay, hypotonia, developmental retardation, epilepsy, height-weight delay
		71025196–77807140 (#8)	6.78	257363	15-year-old	F	Mental retardation, speech delay, hypotonia, developmental retardation, epilepsy, autistic behaviour, height-weight delay, facial dysmorphy, skeletal malformations
		70950015–83592446 (#9)	12.64	258216	4.5-year-old	M	Mental retardation, speech delay, hypotonia, developmental retardation, epilepsy, abnormal brain MRI, height-weight delay, facial dysmorphy, ocular abnormalities
		75091854–81486732 (#10)	6.39	2064	-	M	Intellectual disability, speech delay, hypotonia, developmental retardation, epilepsy, autism, facial dysmorphy, skeletal malformations
		71128855–81486732 (#11)	10.36	2065	-	M	Intellectual disability, speech delay, developmental retardation, hyperactivity, seizure
		71128848–82257009 (#12)	11.13	249623	-	-	Intellectual disability, speech delay, developmental retardation, hyperactivity, seizure
		71128848–79023977 (#13)	7.90	249451	-	-	Intellectual disability, proportionate short stature, strabismus
Lal et al. (2015)	1	77112251–77303533	0.58	-	2-3-year-old	M	Intellectual disability, epilepsy, behavioral problems with aggressive features
Genesio et al. (2015)	1	72803705–80243747	7.44	-	10-year-old	F	Severe intellectual disability, epilepsy, global developmental delay, dysregulation of platelet aggregation, dysmorphisms, genitalia malformations and hypothyroidism
Tuğ et al. (2018)	1	71069763–86333272	15.26	-	22-month-old	M	Intellectual disability, development delay, absent speech, epilepsy, autistic behaviour/behavioral problems, attention deficit, hyperactivity disorder and dysmorphic craniofacial features (relative macrocephaly, midface flattening, frontal bossing, sparse medial eyebrows, hypertelorism, broad base to the nose, smooth philtrum, large and open mouth, thin upper lip, operation scar on the lip, wide spaced teeth, and posterior rotated ears)
De et al. (2023)	1	75505408–83868435	8.36	-	7-year-old	M	Global developmental delay, intellectual disability, autistic behaviour, seizures and facial dysmorphism
DECIPHER	14	77206264–77240837	0.03	254951	-	F	Epicanthus, fragile nails, full cheeks, high palate, intellectual disability, microcephaly, myopia, seizure, strabismus, vertical nystagmus, obsolete joint laxity
		71069563–76223433	5.15	258926	-	F	Cognitive impairment, spotty hyperpigmentation, strabismus
		72464822–75835811	3.37	281725	-	M	Atonic seizure, delayed speech and language development, downslanted palpebral fissures, generalized-onset seizure, high palate, lactose intolerance, short lingual frenulum
		76474486–81651005	5.18	288874	-	-	Intellectual disability, seizure
		70984484–76936284	5.95	289387	-	M	Global developmental delay
		71031677–76816695	5.79	290187	-	-	Atypical behavior, intellectual disability
		70984481–79549501	8.57	322547	-	F	Generalized-onset seizure, mild intellectual disability, obsolete absence seizures with special features
		76792356–77124539	0.33	326506	-	M	Intellectual disability, specific learning disability
		73307216–80890936	7.58	333517	-	M	Absent speech, absent vas deferens, autism, global developmental delay, hydrocele testis, moderately short stature, nystagmus, short femur
		76698559–83614583	6.92	353804	-	F	Abnormal facial shape, delayed speech and language development, global developmental delay, strabismus
		74422207–77096282	2.67	359571	-	F	Autism, specific learning disability
		70901814–76555130*	5.65	433930	-	M	Intellectual disability, migraine, seizure
		71033538–76540293*	5.51	480959	-	F	Autism, global developmental delay, seizure
		77068828–78138272*	1.07	547925	-	F	Moderate intellectual disability, microcephaly, ptosis, seizure, short stature
Our patients	3	74870591–77222652 (Ⅰ-2)	2.35	-	-	F	Intellectual disability
		74870591–77222652 (Ⅱ-2)	2.35	-	-	F	Slight intellectual disability accompanied with abnormal electromagnetic waves
		74870591–77222652 (Ⅲ-2)	2.35	-	2-year-old	F	Epilepsy, intellectual disability, speech impairment, delayed motor development, autism

“*” has been lifted over in GRCh37 (LiftOver from USCS website, https://genome.ucsc.edu/cgi-bin/hgLiftOver accessed on 6 June 2025).

The location of deletion region in the patients we detected is different from before, and it does not cover the minimum overlapping region of 750 Kb as previously reported (Boudry-Labis et al., 2013). However, there are similar clinical phenotypes to previous reports, indicating that 9q21.13 microdeletion syndrome is not only caused by a specific gene or genomic region. We should analyze the specific deletion specifically. The deletion region in our study contains 6 protein coding genes, namely, *ALDH1A1*, *ANXA1*, *GDA*, *RORB*, *TMC1* and *ZFAND5*. Of which, *RORB* and *TMC1* are OMIM Morbid genes.

*RORB*, as a nuclear orphan receptor, can regulates neuronal patterns during cortical development and may be involved in neuronal cell differentiation (Jabaudon et al., 2012; Rossini et al., 2011). *Rorb−/−* mice exhibited damage in several neural reflexes, retinal degeneration, altered circadian activity, increased exploratory activity, less depressive-like behavior, reduced anxiety behavior, and ataxia, indicating *RORB* may be involved in sensory processing and emotional regulation (Masana et al., 2007). In addition, recent studies from SNP tagging and genotyping manifest that there is a correlation between *RORB* and bipolar disorder (McGrath et al., 2009; Partonen, 2012). Sadleir et al. reported 11 affected individuals from 4 families with inherited *RORB* variants with an overlap of occipital epilepsy and photosensitive genetic generalised epilepsy (GGE) (Sadleir et al., 2020). Each family has one novel variant, 3 families with missense variants and one with exon 3 deletion of *RORB*, which located in key evolutionary conserved domains and were all predicted to be pathogenic by *in silico* tools, and not previously seen in population databases (Sadleir et al., 2020). What’s more, the intragenic microdeletion was predicted to give rise to loss-of-function (Sadleir et al., 2020).

In 2013, a report revealed that *RORB* has been deleted in all 13 cases with 9q21.13 microdeletion syndrome characterised by epilepsy, intellectual disability, speech delay, autism, and moderate facial deformities (Boudry-Labis et al., 2013). In this family, no obvious facial deformities were observed, which may be due to atypical facial features or inconsistent fragments with previous reports, which needs further observation and in-depth research. Gokce-Samar et al. (2024) reported 32 different heterozygous variants in *RORB* carried by 35 patients, including 28 single nucleotide variants or small insertions/deletions and 4 microdeletions; among them, 89% of patients had epileptic seizures, and 85% of patients had varying degrees of intellectual disability. A large-scale European case-control cohort study on GGE identified a microdeletion only containing the exon 1 of *RORB* gene from a male patient with childhood absence epilepsy (Lal et al., 2015), overlapping with the critical region of 9q21.13 microdeletion syndrome (Boudry-Labis et al., 2013). The deleted region we detected contains *ALDH1A1*, *ANXA1*, *GDA*, *TMC1*, *ZFAND5* and the first exon of *RORB* and caused epilepsy and intellectual disabilities, which is consistent with the phenotypes of patient in the case-control study mentioned above. Therefore, we assume that the *RORB* gene plays a crucial role in the 9q21.13 microdeletion. Other evidence suggests that nonsense and missense variations involving the *RORB* gene, as well as various sizes of CNVs, may lead to HI of *RORB*, resulting in common phenotypic features involving intellectual disability and generalised epilepsy (Rudolf et al., 2016).

The above numerous pieces of evidence have shown that the *RORB* is a strong candidate gene for complex neurodevelopmental disorders and also has a strong association with 9q21.13 microdeletion syndrome. As is known, the *RORB* gene is associated with susceptibility to idiopathic generalized epilepsy-15 (EIG15; OMIM #618357), an autosomal dominant disease with possible incomplete penetrance which is characterized by the occurrence of different types of epileptic seizures within the first decade (Rudolf et al., 2016; Dong et al., 2021). In 2016, in a 4-generation French family with EIG15, 4 affected individuals carried a heterozygous nonsense variation in the *RORB* gene which is detected by WES, and confirmed by Sanger sequencing (Rudolf et al., 2016). And this variation segregated with the disease phenotype in this family, which may be a loss-of-function variation (Rudolf et al., 2016). The evidence from Rudolf et al. (2016) strongly indicates that both nonsense and missense variants, and various CNVs in *RORB* may lead to the *RORB* HI, which is almost absent in the control population, generating some related neurodevelopmental phenotypes such as intellectual disability and epilepsy.

*TMC1*, another OMIM Morbid gene, whose variation associated with autosomal dominant deafness-36 (DFNA36; OMIM #606705) and autosomal recessive deafness-7 (DFNB7), also known as DFNB11 (OMIM #600974) (Kurima et al., 2002). None of our patients have a phenotype of hearing loss. In a study of autism rat models, it was found that the retinoic acid synthase ALDH1A1 was downregulated at both mRNA and protein levels in the valproic acid (VPA)-treated offspring (Yuan et al., 2023). Supplementing with retinoic acid improves motor coordination in autism model rats, indicating the role of ADH1A1 in the synthesis of retinoic acid (Yuan et al., 2023). However, There is no relevant OMIM disease information for *ALDH1A1*, as well as *ANXA1*, *GDA* and *ZFAND5*. It require extensive research and case analysis to determine their association with diseases.

In summary, we detected microdeletions at the 9q21.13 in the samples of the proband and her mother using WES technology. Due to the detection limitations of WES in detecting CNVs, we then validated the microdeletions via CMA technology and found that the locations of the microdeletion of the proband, her mother and her grandmother were identical, which was generally consistent with the regions detected by WES. The final assessment of CNV pathogenicity was based on CMA result, i.e., pathogenic. Moreover, CMA testing of amniotic fluid DNA revealed the same variation in the fetus with no clinical phenotypes. Nevertheless, it cannot be guaranteed that it will not develop symptoms after birth, and the severity of its phenotypes cannot be predicted. Given the symptoms of other family members and other reported cases of 9q21.13 microdeletion syndrome, the classification of this CNV is pathogenic. Therefore, it deserves the attention of prenatal diagnostic physicians and genetic counselors. Intellectual disability is a common feature among the affected members in this 3-generation Chinese family, and other clinical phenotypes include epilepsy, speech impairment, delayed motor development, and autism. After reviewing relevant literatures, we speculate that *RORB* has a significant contribution to the clinical phenotypes caused by this microdeletion.

## Data Availability

The data that support the findings of this study have been deposited into CNSA with accession number CNP0007603.
